# The Erk/CREB Signaling Axis Mediates G-CSF-Promoted G-MDSC Survival in the Peripheral Blood of Melanoma-Bearing Mice

**DOI:** 10.3390/biology15171511

**Published:** 2026-09-03

**Authors:** Yingying Sun, Yan Mo, Boya Xu, Chao Shang, Shu Jiang, Jing Xu, Yueshuang Ke, Xianlu Zeng

**Affiliations:** 1 School of Medicine, Huanghuai University, Zhumadian 463000, China; 2 The Key Laboratory of Molecular Epigenetics of Ministry of Education, Institute of Genetics and Cytology, School of Life Science, Northeast Normal University, Changchun 130024, China

**Keywords:** tumor conditions, G-MDSC persistence, G-CSF, Erk/CREB pathway

## Abstract

Myeloid-derived suppressor cells (MDSCs) are heterogeneous cell populations that promote tumor development by inhibiting innate and adaptive immunity. Under physiological conditions, neutrophils have a short survival time in vivo. However, G-MDSCs accumulate abnormally in most tumor hosts, and the mechanisms underlying G-MDSC persistence remain to be clarified. This study utilized flow cytometric analysis and in vivo assays to elucidate these mechanisms. It was observed that the apoptosis of G-MDSCs in the peripheral blood of mice was reduced under tumor conditions, whereas an increase in granulocyte colony-stimulating factor (G-CSF) promoted the survival of G-MDSCs. Furthermore, G-CSF reduced the apoptosis of G-MDSCs by activating the Erk/CREB pathway. Thus, inhibition of G-CSF/Erk/CREB signaling may promote the apoptosis of G-MDSCs and shorten their persistence under tumor conditions, as well as attenuate tumor progression.

## Simple Summary

Myeloid-derived suppressor cells (MDSCs) are a group of immunosuppressive cells that, rather than eliminating cancer cells, promote tumor progression by suppressing anti-tumor immune responses. In this study, we investigated the mechanisms that regulate G-MDSC survival in a mouse model of melanoma. We found that granulocyte colony-stimulating factor (G-CSF), a cytokine frequently elevated in the tumor microenvironment, provides critical survival signals to MDSCs. Mechanistically, G-CSF activates the ERK/CREB signaling pathway in MDSCs, thereby enhancing their resistance to apoptosis and promoting their persistence within tumors. Targeting this signaling axis increased G-MDSC apoptosis and consequently suppressed tumor development. These findings identify the G-CSF/ERK/CREB pathway as a potential therapeutic target for disrupting tumor-mediated immune suppression.

## 1. Introduction

Myeloid-derived suppressor cells (MDSCs) are heterogeneous, immature immunosuppressive cells that are produced in response to injury, infection, or tumor formation in the body. In the tumor microenvironment—a significant pathological setting closely related to high mortality [1,2]—several inflammatory mediators contribute to the accumulation and immunosuppression of MDSCs [3]. The mechanisms through which MDSCs exert immunosuppressive functions have been well elucidated. Namely, they include the expression of arginase-1 (Arg1), inducible nitric oxide synthase (iNOS) [4,5,6], TGF-β [7,8,9], IL-10 [10], and COX2 [11,12,13], sequestration of cysteine [14], decreased L-selectin expression by T cells [15], as well as induction of Tregs [16].

MDSCs comprise granulocytic MDSCs (G-MDSCs), characterized by a granulocytic phenotype, and mononuclear MDSCs (M-MDSCs), which exhibit a monocytic phenotype. In mice, G-MDSCs and M-MDSCs are defined as CD11b+Ly6C^low^Ly6G+ and CD11b^+^Ly6C^hi^Ly6G^-^, respectively. In humans, G-MDSCs are CD14-CD11b+CD33+CD15+ cells, whereas M-MDSCs comprise CD14+HLA-DR- or CD11b+CD14-CD33+CD15- cells. Studies have shown that the phenotype and functional properties of MDSCs may vary depending on the type of tumor and the animal model used [4,17,18]. The distribution of MDSC subpopulations also exhibits significant differences among patients with different types of cancer. For instance, G-MDSCs are primarily detected in patients with renal cell carcinoma, non-small cell lung cancer, and pancreatic adenocarcinoma [19,20,21], whereas M-MDSCs predominate in glioblastoma and hepatocellular carcinoma [22,23]. In addition, existing data reveal that MDSCs have distinct functions in peripheral lymphoid organs and tumor sites. The former is predominantly composed of G-MDSCs, which have relatively weak suppressive activity and primarily mediate T-cell tolerance by regulating tumor-specific immune responses [24].

The definition of G-MDSCs as a distinct population has aroused controversy among some researchers owing to their similarity in phenotypes with some types of neutrophils [25]. In several reports, neutrophils with immunosuppressive functions are called N2, and antitumor neutrophils are called N1 [26]. N1 cells are more likely to represent activated neutrophils, whereas N2 cells are pathologically activated neutrophils, namely G-MDSCs [27,28,29]. It is well known that neutrophils have a short lifespan under normal physiological conditions. However, tumor cells and stromal cells can secrete a variety of cytokines to induce the accumulation and persistence of G-MDSCs under tumor conditions [30]. It has been suggested that the survival of G-MDSCs under tumor conditions is likely to be a prerequisite for immunosuppression [31]. Nevertheless, the underlying mechanisms of G-MDSC persistence in tumor-bearing hosts remain unclear.

Granulocyte colony-stimulating factor (G-CSF) is a major hematopoietic cytokine that regulates granulopoiesis and is widely used in the treatment or prevention of neutropenia [32,33]. G-CSF is also an effective stimulator that induces neutrophil release from bone marrow [34]. Under tumor conditions, the level of G-CSF in the serum of mice increases significantly, which is reportedly related to immunosuppression [35,36]. Whether G-CSF affects the persistence of G-MDSCs under tumor conditions and the specific mechanism remain to be further elucidated.

Extracellular-signal-regulated kinases 1 and 2 (Erk1/2) are members of the mitogen-activated protein kinase (MAPK) family that regulate many cell functions [37]. Specifically, Erk1/2 phosphorylates substrates to control downstream signaling. The cAMP response element binding protein (CREB), which functions as a transcription factor, is downstream of Erk1/2. CREB can regulate a variety of cellular processes, such as proliferation, apoptosis, and metabolism [38]. The present study discussed the role of Erk/CREB in G-MDSC persistence. As the tumor progressed, the level of G-CSF in the serum of mice was upregulated, whereas the apoptosis of G-MDSCs was significantly reduced. Additionally, the mechanism of G-MDSC apoptosis reduction was explored, and the results indicated that the increase in G-CSF under tumor conditions reduced the apoptosis of G-MDSCs and promoted the survival of G-MDSCs by activating the Erk/CREB signaling pathway. Therefore, inhibition of G-CSF/Erk/CREB signaling may promote G-MDSC apoptosis and delay tumor progression.

## 2. Materials and Methods

### 2.1. Mice and Cell Lines

C57BL/6J mice (8–10 weeks old) were purchased from Beijing Vital River Laboratory Animal Technology Co., Ltd. (Beijing, China). Mouse melanoma B16F10 cells (1 × 10^6^) were subcutaneously injected into the back of female mice. The tumor volume was calculated as: V = (length × width^2^) × 0.5. All mice were housed under specific pathogen-free conditions. B16F10 cells (ATCC CRL-6475, RRID: CVCL_0159) and human embryonic kidney HEK-293T cells (ATCC CRL-3216, RRID: CVCL_0063) were purchased from the American Type Culture Collection (ATCC, Manassas, VA, USA). All cells were cultured in DMEM supplemented with 10% heat-inactivated FBS (HyClone, Logan, UT, USA) and 1% penicillin/streptomycin. All cells were routinely validated for the absence of Mycoplasma infection using the LookOut Mycoplasma PCR Detection Kit (Sigma, St. Louis, MO, USA) and were used for experimentation within 10 passages.

### 2.2. Reagents and Antibodies

Recombinant murine G-CSF (cat. No. 250-05) was purchased from PeproTech (Rocky Hill, NJ, USA). Directly conjugated anti-mouse mAbs CD11b-FITC (clone M1/70, cat. No. 553310), CD45-PE/Cy7 (clone 30-F11, cat. No. 552848), Ly6G-APC/Cy7 (clone 1A8, cat. No. 560600), and Ly6C-PE (clone AL-21, cat, No. 560592) were purchased from BD Pharmingen (San Jose, CA, USA). The Annexin V-APC apoptosis analysis kit (cat. No. AO2001-11P-G) was obtained from Sungene (Tianjin, China). Anti-p44/42 MAPK (Erk1/2, clone L34F12, cat. No. 4696), anti-p-p44/42 MAPK (Erk1-Tyr204/Erk2-Tyr187, clone D1H6G, cat. No. 5726), anti-CREB (clone 86B10, cat. No. 9104) and anti-p-CREB (Ser133, clone 1B6. cat. No. 9196) Abs were purchased from Cell Signaling Technology (Danvers, MA, USA). U0126 (cat. No. HY-12031A) and 666-15 (cat. No. HY-101120) were purchased from Med Chem Express (Monmouth Junction, NJ, USA).

### 2.3. Flow Cytometry

Peripheral blood leukocytes obtained after ammonium chloride-based erythrocyte lysis were stained on ice for 30 min with fluorochrome-conjugated antibodies. Apoptosis was determined with Annexin V-APC apoptosis analysis kit (Sungene Biotech, Tianjin, China; cat. No. AO2001-11P-G), and fluorescence was measured using a FACSCanto II flow cytometer (BD Biosciences, San Jose, CA, USA), and the data were analyzed with FlowJo v10 software.

### 2.4. Cell Isolation

Bone marrow was flushed from mouse femurs and tibiae using PBS supplemented with 2% FBS. Single cell suspensions were generated with 1 mL syringes, and erythrocytes were removed using an ammonium chloride lysis buffer. The cells were then labeled with biotinylated anti-Ly6G antibody (Miltenyi Biotec, Bergisch Gladbach, Germany; clone REA526, cat. No. 130-107-911) and streptavidin-coated microbeads (Miltenyi Biotec, Bergisch Gladbach, Germany; cat. No. 130-048-101), and then separated on MACS columns (Miltenyi Biotec, Bergisch Gladbach, Germany).

### 2.5. RNA Extraction and qPCR

Total RNA was extracted from cells using TRIzol (Takara, Kusatsu, Shiga, Japan), and cDNA was synthesized using reverse transcriptase (Thermo Fisher, Waltham, MA, USA). The cDNA was used as a template for qPCR with Power SYBR Green PCR Master Mix (Thermo Fisher) on a StepOne Plus Real-Time PCR System (Applied Biosystems, Foster City, CA, USA). Primer sequences are detailed in Supplementary Table S1.

### 2.6. Enzyme-Linked Immunosorbent Assay (ELISA)

The concentration of G-CSF in the serum was determined with ELISA kits (eBioscience, San Diego, CA, USA) according to the instructions provided by the manufacturer.

### 2.7. Construction of Lentiviral Expression Plasmid and Gene Transfection

Synthesized short hairpin (sh) RNAs were cloned into the vector pLVTHM and co-transfected with psPAX2 and pMD2.G into HEK-293T cells using PEI. To knock down G-CSF in vivo, concentrated viral supernatants were injected intrapleurally into two-week tumor-bearing mice every other day for four injections in total. shRNA sequences are detailed in Supplementary Table S2.

### 2.8. Western Blot

Cells were lysed in RIPA buffer supplemented with protease inhibitors. Protein samples (10 μg) were resolved by SDS-PAGE and transferred onto nitrocellulose membranes. After blocking, membranes were incubated overnight at 4 °C with primary antibodies (diluted 1:1000), and then washed and incubated with goat anti-rabbit-HRP antibodies or goat anti-mouse-HRP secondary antibodies. Immunoreactive bands were visualized using a chemiluminescent substrate. The original Western blotting images are presented in the Supplementary Figure S1.

### 2.9. Tumor Metastasis Assay In Vivo

C57BL/6J mice were injected intravenously with 2 × 10^5^ B16F10 cells, and lung metastatic nodules were counted after 2 weeks. To determine the effects of the Erk/CREB pathway, U0126 (5 mg/kg, Med Chem Express, Monmouth Junction, NJ, USA; cat. No. HY-12031A) or 666-15 (5 mg/kg, Med Chem Express, Monmouth Junction, NJ, USA; cat. No. HY-101120) was administered intraperitoneally at the time of tumor injection and again on days 2 and 4.

### 2.10. Statistical Analysis

For in vivo experiments, each mouse was considered as one experimental unit. For in vitro experiments, each independent cell preparation derived from a separate set of four mice was treated as one experimental unit, and technical replicates (performed in triplicate per sample) were averaged within each independent experiment. Statistical analysis was performed using GraphPad. The results are presented as mean ± SEM and analyzed using an unpaired *t*-test or a one- or two-way ANOVA (analysis of variance) followed by a post hoc test. Differences were considered statistically significant if *p* < 0.05 (* *p* < 0.05; ** *p* < 0.01; and *** *p* < 0.001).

## 3. Results

### 3.1. Establishment of a Subcutaneous Melanoma Model

**Figure 1:**
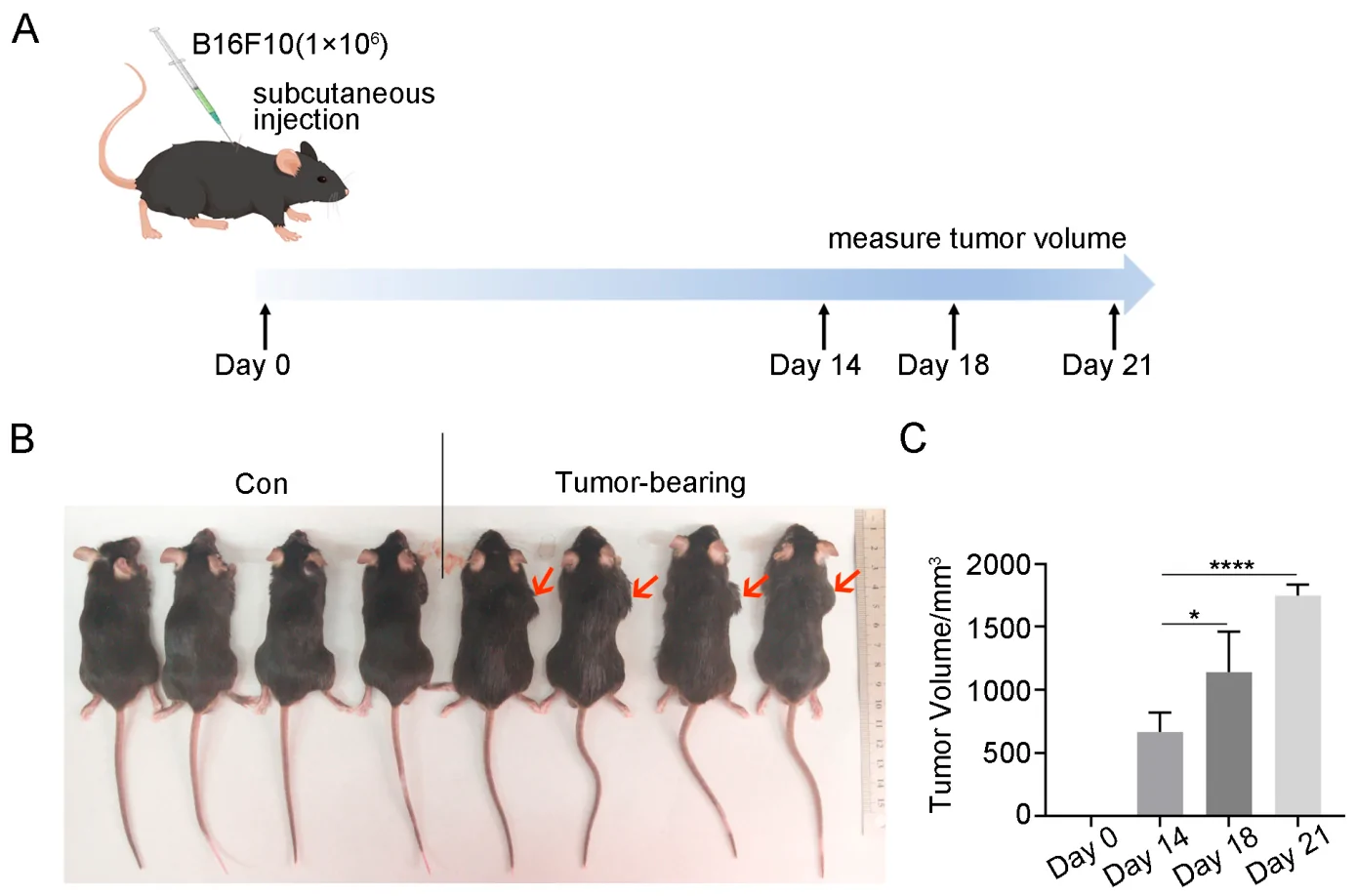
Establishment of the tumor model. (**A**) Experimental design and timeline of subcutaneous melanoma transplantation in mice. (**B**) Representative images of subcutaneous melanoma tumors excised from mice on day 18 following injection of B16F10 cells (n = 4 mice per group). Red arrows indicate the tumor. (**C**) Tumor volume measured at indicated time points following injection of B16F10 cells (n = 4 mice per group). * *p* <  0.05, **** *p* <  0.0001.

A subcutaneous melanoma model was first established by injecting B16F10 cells into C57BL/6J mice (Figure 1A). As expected, these mice gradually developed palpable subcutaneous tumors after inoculation, and representative images of tumor-bearing mice at day 18 post-inoculation were shown in Figure 1B. Tumor volumes were measured at the indicated time points (Figure 1C), revealing a progressive growth pattern compared to control mice.

### 3.2. Apoptosis of G-MDSCs Decreased in the Peripheral Blood of Tumor-Bearing Mice

**Figure 2:**
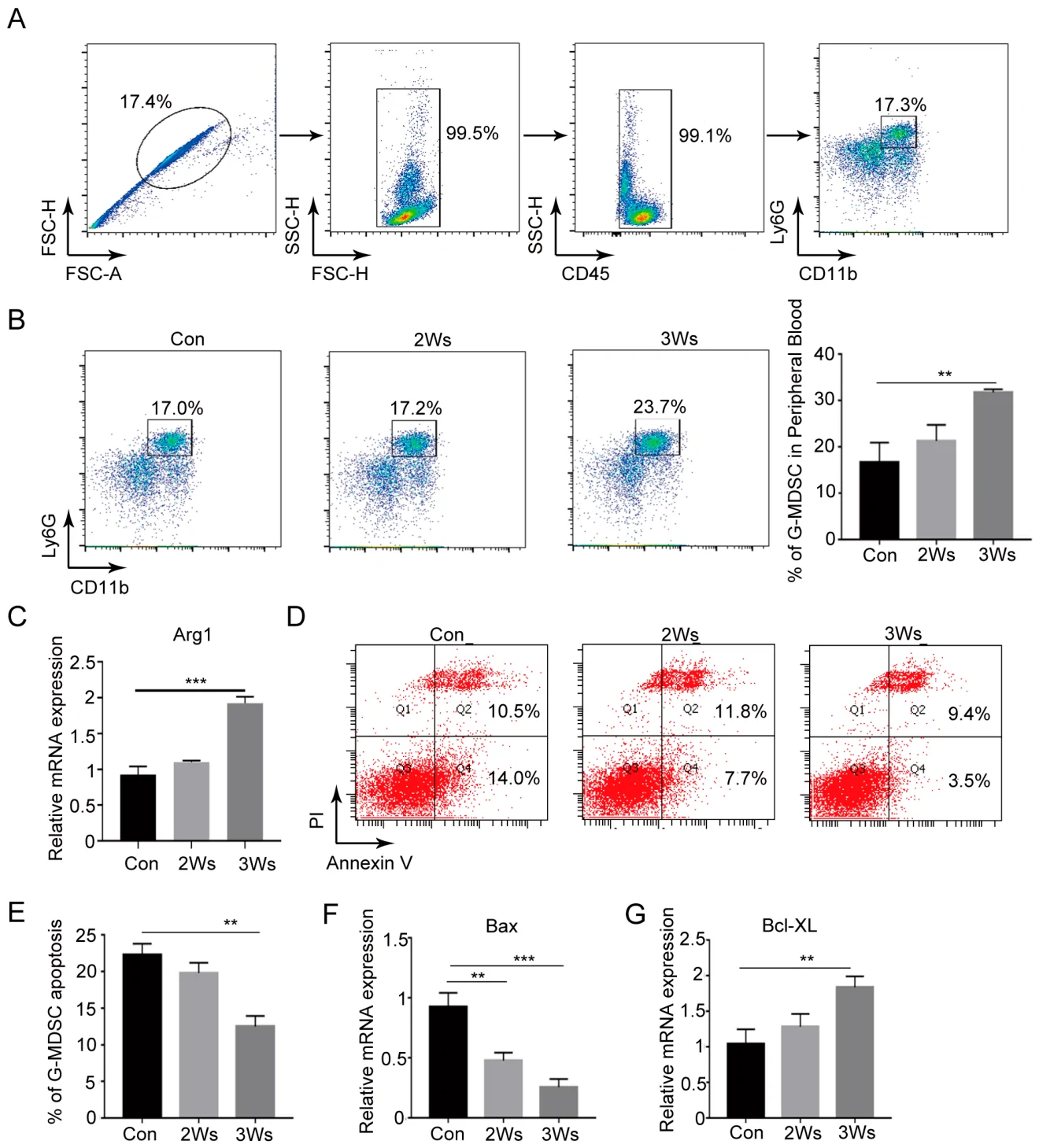
The apoptosis of G-MDSCs in the peripheral blood of mice decreases under tumor conditions. (**A**) Identification of G-MDSCs (CD45+CD11b+Ly6G+) via flow cytometry. (**B**) Proportion of G-MDSCs in the peripheral blood of mice bearing tumors at different stages analyzed by flow cytometry (n = 6 mice per group). (**C**) Expression levels of Arg1 in G-MDSCs of peripheral blood analyzed via qPCR at different time points of tumor progression (n = 6 mice per group). (**D**,**E**) Apoptosis of G-MDSCs in the peripheral blood of mice bearing tumors at different stages analyzed by flow cytometry (n = 6 mice per group). (**F**,**G**) Expression levels of Bax and Bcl-xL in G-MDSCs from the peripheral blood analyzed via qPCR at different time points of tumor progression (n = 6 mice per group). ** *p* <  0.01, *** *p* <  0.001.

To investigate whether the survival of G-MDSCs contributed to their prevalence, a tumor mouse model was established by subcutaneously injecting the melanoma cell line B16-F10. Subsequently, the number of G-MDSCs in the peripheral blood was detected via flow cytometry (Figure 2A), and the results revealed that G-MDSCs accumulated abnormally in the peripheral blood of tumor-bearing mice (Figure 2B). Additionally, the expression of their immunosuppressive gene Arg1 was also significantly upregulated (Figure 2C). To investigate the contribution of tumor conditions to the abnormal accumulation of G-MDSCs in the peripheral blood, the proliferation of G-MDSCs in the peripheral blood was detected by labeling with Ki67. It was observed that the proliferation of G-MDSCs in the peripheral blood of tumor-bearing mice did not change significantly compared with that of G-MDSCs in the peripheral blood of normal mice (Figure S2). Furthermore, to explore whether tumor conditions contributed to the accumulation of G-MDSCs by preventing these cells from undergoing apoptosis, the apoptosis of G-MDSCs was detected in the peripheral blood by labeling with Annexin V and PI (Figure 2D). The results indicated that the apoptosis of G-MDSCs in the peripheral blood of tumor-bearing mice decreased under tumor conditions (Figure 2E). Subsequently, G-MDSCs from the peripheral blood of tumor-bearing mice were sorted at different stages using magnetic beads, and the expression of the proapoptotic gene *Bax* as well as the antiapoptotic gene *Bcl-xL*, which represents a well-documented anti-apoptotic regulator enabling tumor-associated MDSCs to resist apoptosis and persist in the tumor microenvironment [39], was analyzed via qPCR. Consequently, the *Bax* mRNA levels were revealed to be significantly decreased, whereas the *Bcl-xL* mRNA levels were significantly increased in G-MDSCs from the peripheral blood of tumor-bearing mice (Figure 2F,G). These results suggest that tumor conditions promote G-MDSC survival in the peripheral blood of mice by reducing apoptosis.

### 3.3. G-CSF Promotes the Survival of G-MDSCs Under Tumor Conditions

**Figure 3:**
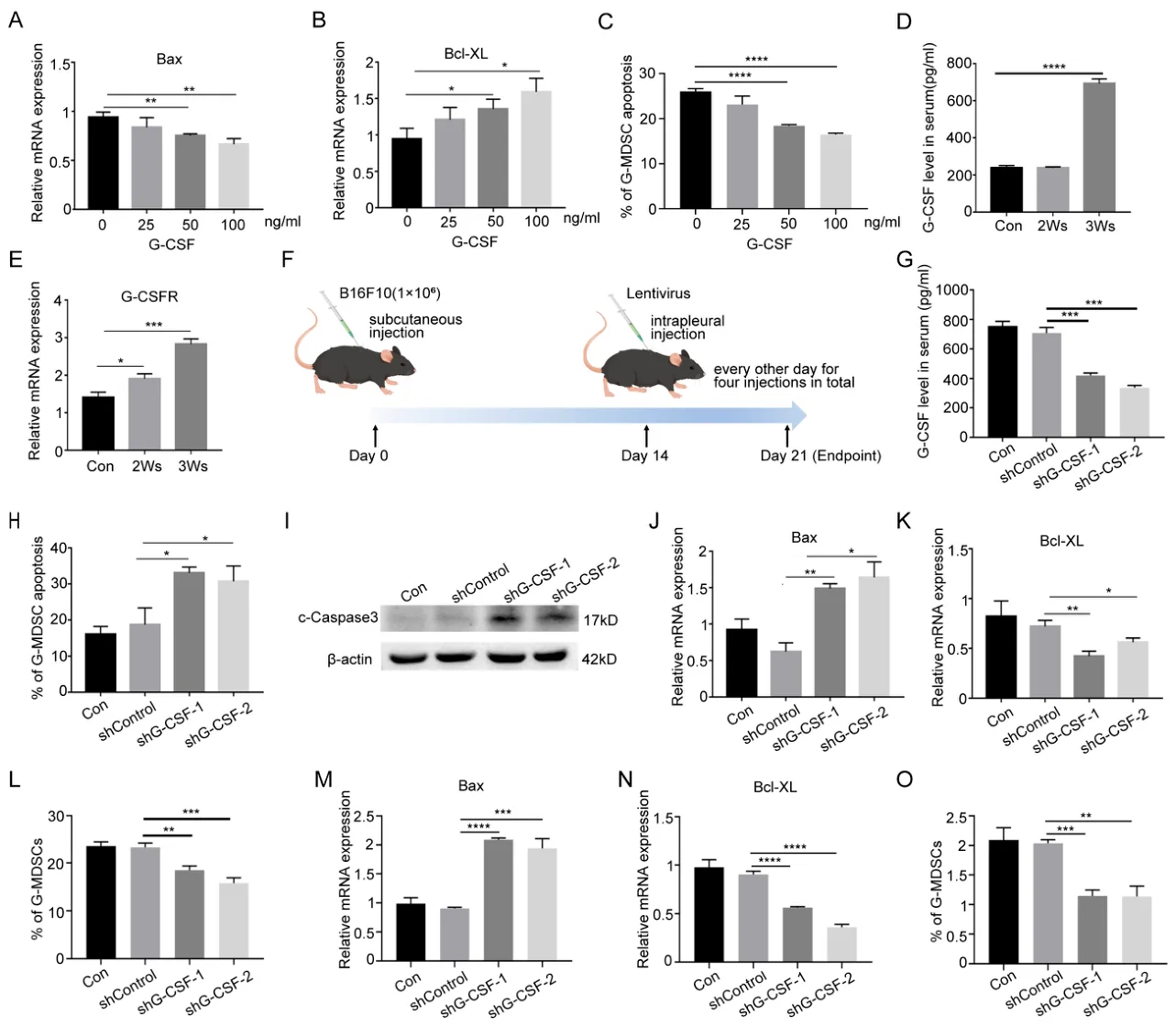
G-CSF promotes the survival of G-MDSCs under tumor conditions. (**A**,**B**) Expression levels of Bax and Bcl-xL in G-MDSCs from the bone marrow of normal mice analyzed via qPCR after incubation with different concentrations of G-CSF for 2 h. (**C**) Apoptosis of G-MDSCs from the bone marrow of normal mice analyzed via flow cytometry after incubation with different concentrations of G-CSF for 24 h. (**D**) Level of G-CSF in the serum of tumor-bearing mice at different stages analyzed by ELISA (n = 6 mice per group). (**E**) Level of G-CSF receptor in G-MDSCs in peripheral blood analyzed via qPCR at different time points (n = 6 mice per group). (**F**) Experimental design and timeline for lentivirus injection into tumor-bearing mice. (**G**) Level of G-CSF analyzed by ELISA after the mice were lentivirally infected with shControl, shG-CSF1, or shG-CSF2 (n = 6 mice per group). (**H**) Apoptosis of G-MDSCs in the peripheral blood of tumor-bearing mice analyzed via flow cytometry after the mice were lentivirally infected with shControl, shG-CSF1, or shG-CSF2 (n = 6 mice per group). (**I**) Level of cleaved caspase-3 in G-MDSCs of peripheral blood analyzed by Western blot after the mice were lentivirally infected with shControl, shG-CSF1, or shG-CSF2. (**J**,**K**) Expression levels of Bax and Bcl-xL in G-MDSCs of peripheral blood analyzed via qPCR after the mice were lentivirally infected with shControl, shG-CSF1, or shG-CSF2. (**L**) Proportion of G-MDSCs in the peripheral blood of mice bearing tumors analyzed by flow cytometry after the mice were lentivirally infected with shControl, shG-CSF1, or shG-CSF2 (n = 6 mice per group). (**M**,**N**) Expression levels of Bax and Bcl-xL in G-MDSCs of tumor tissue analyzed via qPCR after the mice were lentivirally infected with shControl, shG-CSF1, or shG-CSF2 (n = 6 mice per group). (**O**) Proportion of G-MDSCs in the tumor tissue of mice bearing tumors analyzed by flow cytometry after the mice were lentivirally infected with shControl, shG-CSF1, or shG-CSF2 (n = 6 mice per group). * *p* <  0.05, ** *p* <  0.01, *** *p* <  0.001, **** *p* <  0.0001.

Our previous work reported that CXCL10, CXCL13, and G-CSF levels were increased in the serum of tumor-bearing mice [40]. To further explore the relationship between these factors and G-MDSC survival in the present study, G-MDSCs from normal mouse bone marrow were stimulated with different concentrations of these factors in vitro. The results suggested that G-CSF inhibited the expression of *Bax* and promoted the expression of *Bcl-xL* (Figure 3A,B), whereas CXCL10 and CXCL13 did not affect the expression of apoptosis-related genes in G-MDSCs (Figure S3A–F). Furthermore, apoptosis assays confirmed that G-CSF treatment significantly inhibited G-MDSC apoptosis (Figure 3C). As the tumor progressed, the level of G-CSF in serum gradually increased (Figure 3D). The G-CSF receptor on the surface of G-MDSCs in the peripheral blood of mice was also gradually upregulated (Figure 3E). Subsequently, a lentiviral vector stably interfering with G-CSF was constructed for in vivo experiments (Figure 3F), and the efficiency of lentiviral interference was detected (Figure 3G). After G-CSF intervention in mice, the apoptosis of G-MDSCs was significantly increased, and caspase-3 (a key executor of apoptosis) [41] expression also increased noticeably (Figure 3H,I). In addition, the expression of *Bax* increased, whereas the expression of *Bcl-xL* decreased (Figure 3J,K). Accordingly, the number of G-MDSCs in peripheral blood was significantly reduced (Figure 3L). Similar apoptotic gene expression trends and a reduction in G-MDSC number were also observed in tumor tissues (Figure 3M–O). These results suggest that G-CSF upregulation reduces apoptosis and promotes the survival of G-MDSCs in the peripheral blood of tumor-bearing mice.

### 3.4. G-CSF Promotes the Survival of G-MDSCs by Activating Erk

**Figure 4:**
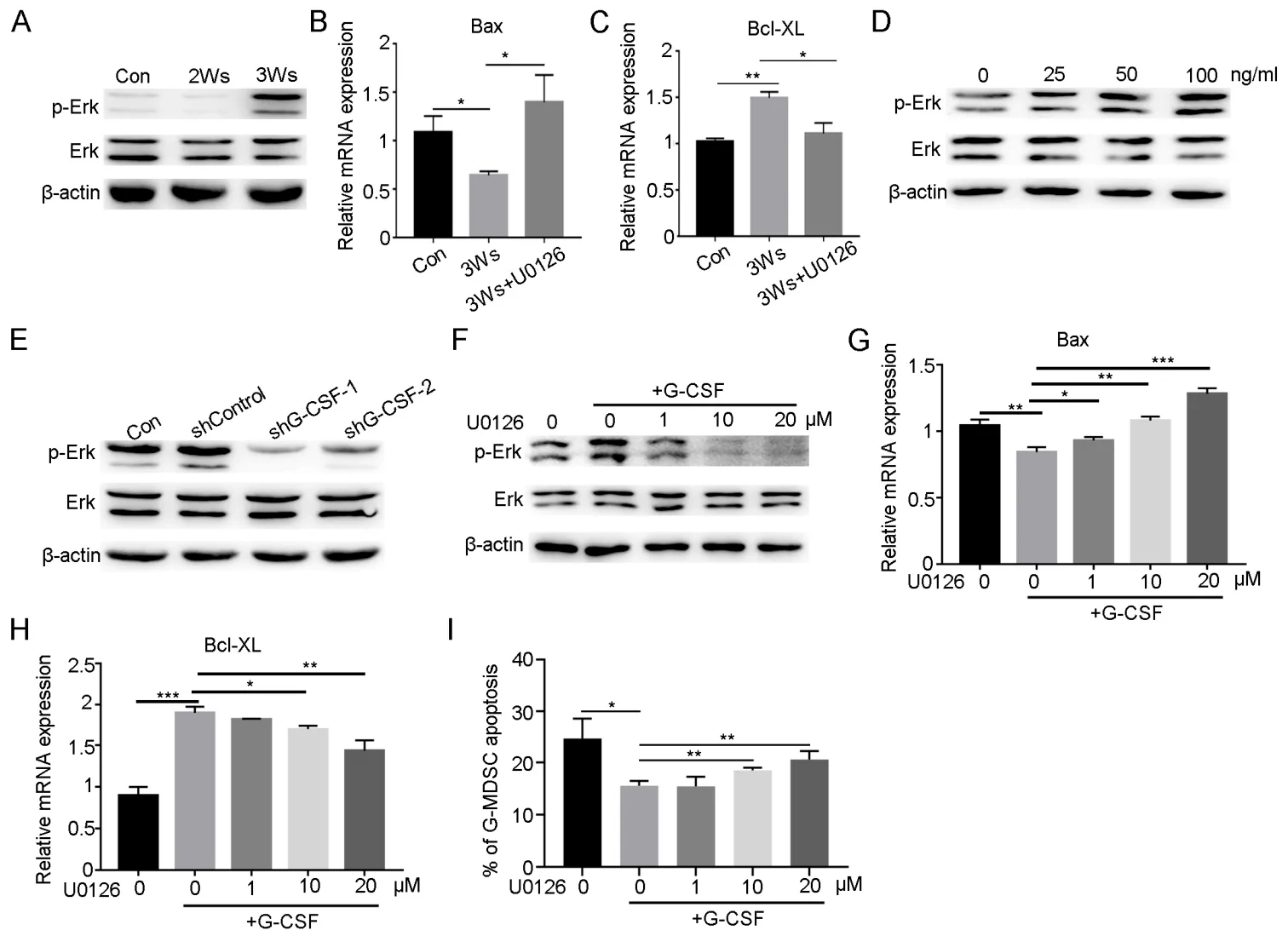
G-CSF activates Erk to promote the survival of G-MDSCs. (**A**) Levels of Erk and p-Erk in G-MDSCs of peripheral blood at different stages of tumor progression analyzed by Western blot. (**B**,**C**) Expression levels of Bax and Bcl-xL in G-MDSCs in peripheral blood analyzed via qPCR after injecting U0126 into tumor-bearing mice. (**D**) Levels of Erk and p-Erk in G-MDSCs analyzed by Western blot after incubation with different concentrations of G-CSF for 2 h. (**E**) Levels of Erk and p-Erk in G-MDSCs analyzed by Western blot after the mice were lentivirally infected with shControl, shG-CSF1, or shG-CSF2. (**F**) Levels of Erk and p-Erk in G-MDSCs in the bone marrow of normal mice detected by Western blot following in vitro culture with G-CSF (50 ng/mL) and different concentrations of U0126 for 2 h. (**G**,**H**) Expression levels of Bax and Bcl-xL in G-MDSCs from the bone marrow of normal mice analyzed via qPCR after culturing in vitro with G-CSF (50 ng/mL) and different concentrations of U0126 for 2 h. (**I**) Apoptosis of G-MDSCs from the bone marrow of normal mice analyzed via flow cytometry after culturing in vitro with G-CSF (50 ng/mL) and different concentrations of U0126 for 24 h. * *p* <  0.05, ** *p* <  0.01, *** *p* <  0.001.

The MAPK/Erk pathway is an important signal transduction pathway that transduces exogenous signals from the cell surface to the nucleus. This pathway can be activated by different stimuli—such as cytokines, neurotransmitters, and hormones—which is followed by a three-stage enzymatic cascade reaction and activation of Erk [42]. The MAPK/Erk pathway is reportedly associated with cell proliferation, differentiation, migration, senescence, and apoptosis [43,44,45]. To explore the mechanism underlying the decrease in G-MDSC apoptosis under tumor conditions, G-MDSCs were isolated from the peripheral blood of tumor-bearing mice at different stages using magnetic beads, and the activity of Erk was detected. The results indicated that the phosphorylation of Erk1/2 in G-MDSCs was upregulated under tumor conditions (Figure 4A). To further investigate whether the activation of Erk in G-MDSCs was related to the reduction in apoptosis, U0126 (an inhibitor of Erk) was injected into the mice. It was observed that U0126 upregulated the expression of the apoptotic gene *Bax* and downregulated the expression of the antiapoptotic gene *Bcl-xL*. This indicates that U0126 promoted the apoptosis of G-MDSCs in the peripheral blood of tumor-bearing mice (Figure 4B,C). Based on the above findings, we hypothesized that a relationship exists between G-CSF and Erk1/2 in G-MDSCs. This hypothesis was tested by stimulating G-MDSCs from the bone marrow of normal mice using G-CSF, in order to detect the phosphorylation of Erk1/2 in vitro. The results suggested that G-CSF increased the phosphorylation of Erk in G-MDSCs (Figure 4D). The phosphorylation of Erk1/2 in G-MDSCs could be reduced by injecting lentivirus that interferes with G-CSF into tumor-bearing mice (Figure 3E). Subsequently, the Erk1/2 inhibitor U0126 was used in in vitro experiments. Following treatment with U0126, the activation of Erk by G-CSF was attenuated (Figure 4F). U0126 also increased the expression of the proapoptotic gene *Bax* and decreased the expression of the antiapoptotic gene *Bcl-xL* (Figure 4G,H), consequently inducing G-MDSC apoptosis (Figure 4I). These results suggest that the upregulation of G-CSF under tumor conditions can promote the survival of G-MDSCs by activating Erk1/2.

### 3.5. Erk-Dependent CREB Phosphorylation Protects G-MDSCs from Apoptosis

**Figure 5:**
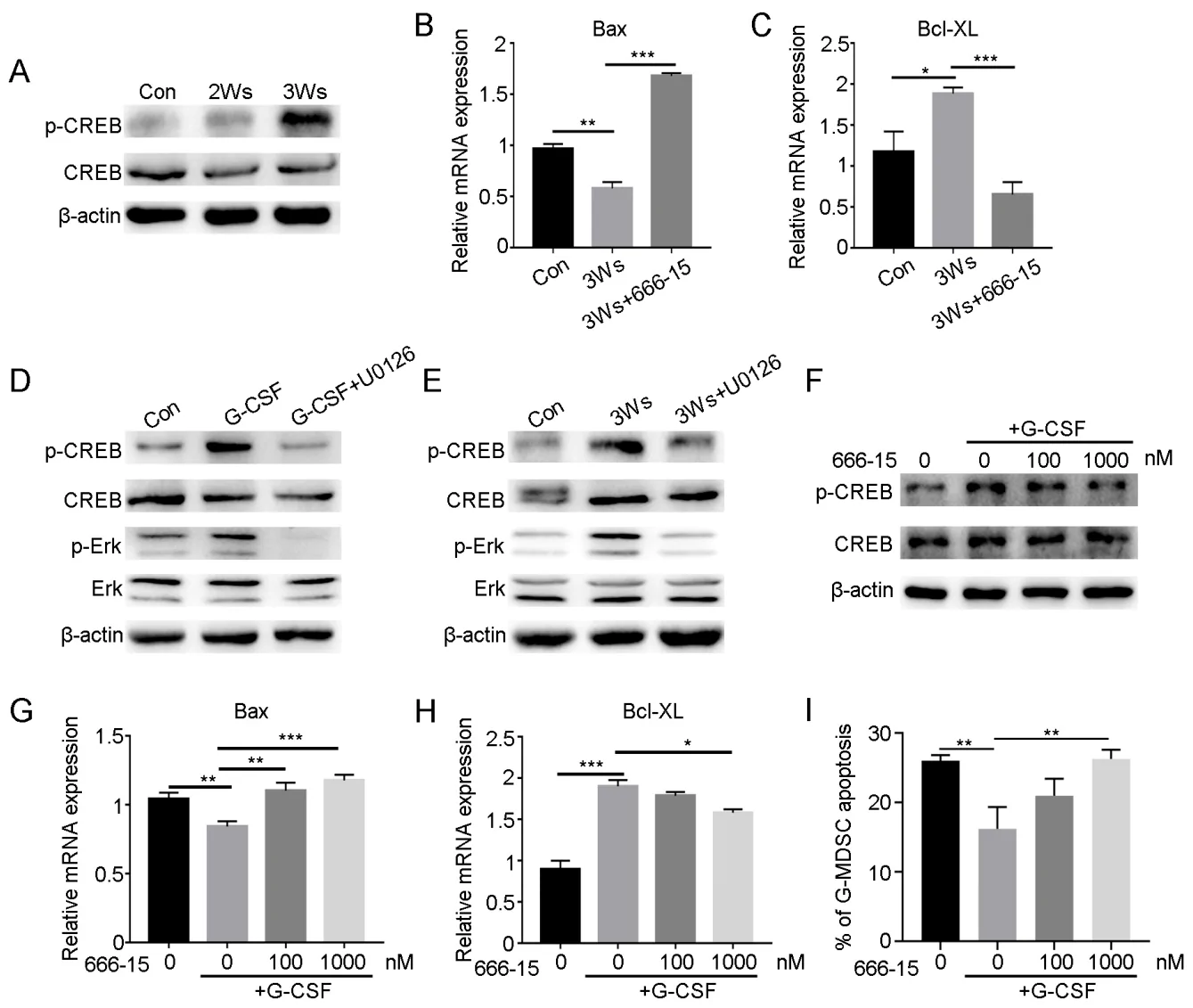
Erk-dependent CREB phosphorylation protects G-MDSCs from apoptosis. (**A**) Levels of CREB and p-CREB in peripheral blood G-MDSCs at different stages of tumor progression detected by Western blot. (**B**,**C**) Expression levels of Bax and Bcl-xL in peripheral blood G-MDSCs analyzed via qPCR after 666-15 was injected into the tumor-bearing mice (n = 6 mice per group). (**D**) Levels of Erk, p-Erk, CREB, and p-CREB detected in G-MDSCs from the bone marrow of normal mice by Western blot after in vitro culture with G-CSF (50 ng/mL) and U0126 for 2 h. (**E**) Levels of Erk, p-Erk, CREB, and p-CREB detected in G-MDSCs of peripheral blood by Western blot after injecting 666-15 into tumor-bearing mice. (**F**) Levels of CREB and p-CREB of G-MDSCs from the bone marrow of normal mice detected by Western blot after in vitro culture with G-CSF (50 ng/mL) and different concentrations of 666-15 for 2 h. (**G**,**H**) Expression levels of Bax and Bcl-xL in G-MDSCs in the bone marrow of normal mice detected via qPCR after in vitro culture with G-CSF (50 ng/mL) and different concentrations of 666-15 for 2 h. (**I**) Apoptosis of G-MDSCs from the bone marrow of normal mice analyzed via flow cytometry after culturing in vitro with G-CSF (50 ng/mL) and different concentrations of 666-15 for 24 h. * *p* <  0.05, ** *p* <  0.01, *** *p* <  0.001.

After mitogen stimulation, Erk is translocated into the nucleus. Therefore, Erk can not only phosphorylate cytoplasmic proteins, but also phosphorylate some nuclear transcription factors—such as c-fos, c-jun, Elk-1, c-myc, and Atf2. It can consequently participate in the regulation of cell proliferation and differentiation [46,47]. CREB is a transcription factor that regulates the expression of various genes involved in apoptosis. The present study observed that the phosphorylation of CREB increased in G-MDSCs from the peripheral blood of tumor-bearing mice (Figure 5A). After treatment with 666-15 (CREB inhibitor), the expression of *Bax* was increased, whereas the expression of *Bcl-xL* in G-MDSCs was significantly reduced (Figure 5B,C), thus indicating that CREB was involved in the survival of G-MDSCs. To delineate the hierarchy between ERK and CREB, ERK1/2 was inhibited, and CREB activation was subsequently assessed. It was deduced that G-CSF-induced CREB phosphorylation was abolished by the ERK1/2 inhibitor U0126, both in vitro as well as in vivo (Figure 5D,E). This result demonstrates that CREB acts downstream of ERK in the G-CSF-driven signaling cascade. To further explore the functional consequences of CREB activation, the CREB inhibitor 666-15 was introduced, following G-CSF stimulation of G-MDSCs. The results demonstrated that CREB inhibition significantly upregulated the expression of the pro-apoptotic gene *Bax* and downregulated the expression of the anti-apoptotic gene *Bcl-xL* (Figure 5F–H), consequently inducing G-MDSC apoptosis (Figure 5I). The above results indicate that G-CSF promotes the survival of G-MDSCs through the Erk/CREB signaling pathway under tumor conditions.

### 3.6. Erk/CREB Inhibitors Delay Tumor Growth and Metastasis by Reducing the Prevalence of G-MDSCs

**Figure 6:**
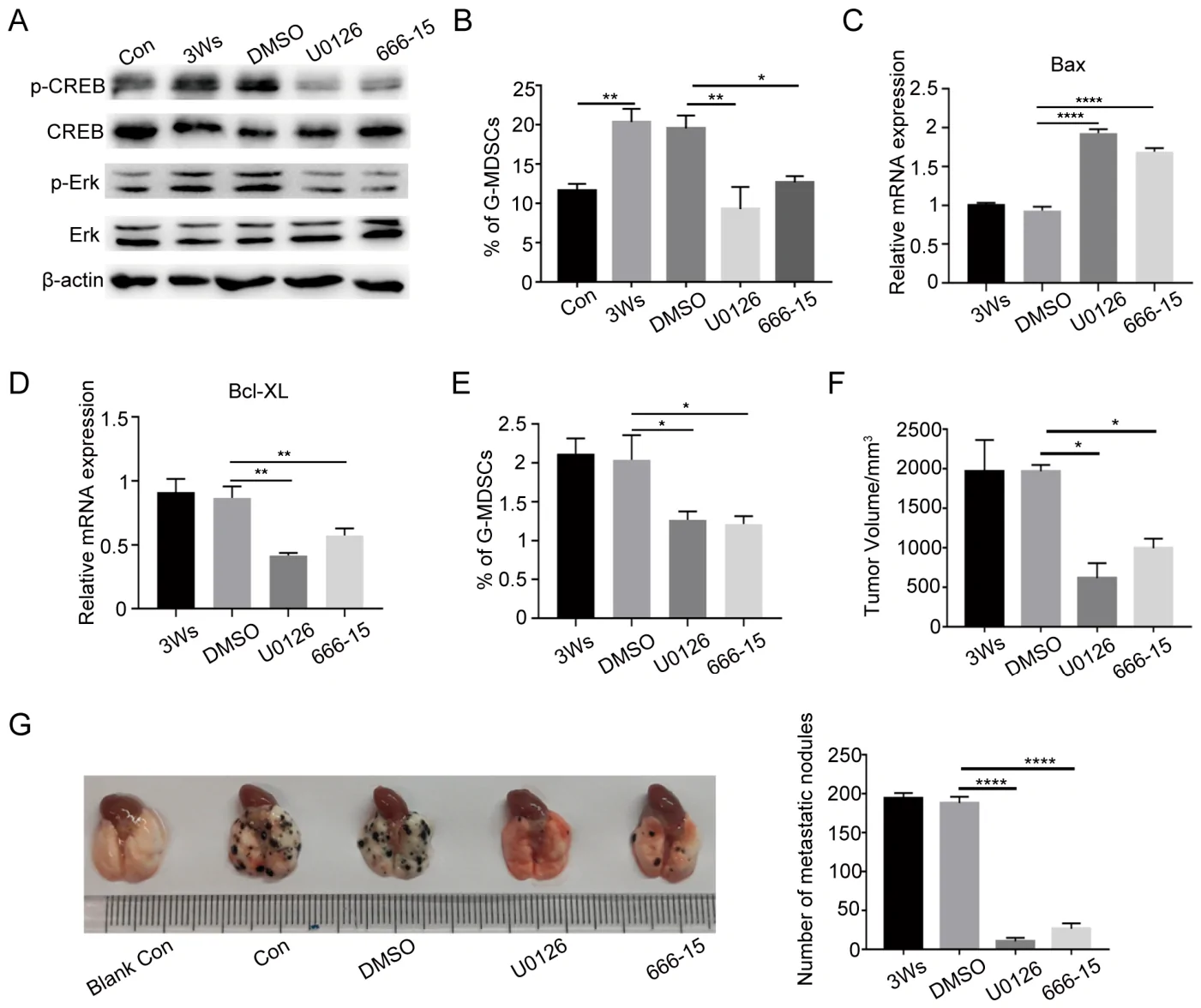
Erk/CREB inhibitors delay tumor growth and metastasis by reducing the number of G-MDSCs. (**A**) Levels of Erk, p-Erk, CREB, and p-CREB detected in G-MDSCs in peripheral blood by Western blot after injecting U0126 or 666-15 into tumor-bearing mice. (**B**) Proportion of G-MDSCs in the peripheral blood of mice bearing tumors at different concentrations analyzed via flow cytometry after U0126 or 666-15 was injected into tumor-bearing mice (n = 6 mice per group). (**C**,**D**) Expression levels of Bax and Bcl-xL of G-MDSCs in the tumor tissue analyzed via qPCR after U0126 and 666-15 were injected into the tumor-bearing mice (n = 6 mice per group). (**E**) Proportion of G-MDSCs in the tumor tissue of mice bearing tumors analyzed by flow cytometry after U0126 and 666-15 were injected into the tumor-bearing mice (n = 6 mice per group). (**F**) Tumor volume measured after injecting U0126 or 666-15 into tumor-bearing mice (n = 4 mice per group). (**G**) Lung metastatic burden detected after injecting U0126 or 666-15 into tumor-bearing mice (n = 4 mice per group); representative images were selected from mice with the median metastatic nodule count for each group. * *p* <  0.05, ** *p* <  0.01, **** *p* <  0.0001.

To determine whether the activation of the Erk/CREB signaling pathway in G-MDSCs promotes tumor growth and metastasis, tumor-bearing mice were treated with Erk/CREB inhibitors via intraperitoneal injection, and the inhibitory efficiency in G-MDSCs was tested (Figure 6A). Then, the number of G-MDSCs was determined via flow cytometry. The results demonstrated that inhibitor treatment significantly reduced the number of G-MDSCs in the peripheral blood compared with that in the control group (Figure 6B). We further examined the expression of apoptotic genes and the number of G-MDSCs in tumor tissue following inhibitor treatment. The results showed that inhibitor treatment upregulated the expression of the pro-apoptotic gene *Bax* (Figure 6C), downregulated the expression of the anti-apoptotic gene *Bcl-xL* (Figure 6D), and reduced the proportion of G-MDSCs (Figure 6E). Furthermore, the inhibitor treatment significantly disrupted tumor growth and metastasis (Figure 6F,G). These results suggest that inhibition of the Erk/CREB signaling pathway delays tumor growth and metastasis by reducing the number of G-MDSCs.

## 4. Discussion

MDSCs are widely defined as immature myeloid cells, which differ from terminally differentiated mature myeloid cells (i.e., macrophages, dendritic cells, or neutrophils) and accumulate in tumor-bearing hosts. In addition to inhibiting the immune response, they directly support tumor growth and metastasis in a variety of ways and are an obstacle to cancer immunotherapy [30,48]. The accumulation and survival of MDSCs may be caused by a variety of mechanisms. MDSCs express the death receptor Fas and undergo apoptosis in response to T cell-expressed FasL [49]. There are also reports that tumor-derived MDSCs have markedly increased levels of phosphorylated STAT3, which prevents their apoptosis [50,51]. The cell death pathways required for the development, survival, and function of the two subtypes of MDSCs are different. The antiapoptotic molecule c-FLIP is necessary for the development of M-MDSCs, and GM-CSF induces MCL-1-related antiapoptotic A1 to further promote their survival. In contrast, the development of G-MDSCs requires a different antiapoptotic molecule, MCL-1 [52]. It has been reported that G-CSF plays a key role not only by regulating the production of neutrophils but also by modulating the activity of these cells [53,54]. In addition, it has been reported that G-CSF can promote immunosuppression under tumor conditions [55,56]. The present study observed that G-CSF was upregulated in the serum of tumor-bearing mice and that excessive G-CSF levels under tumor conditions promoted the survival of G-MDSCs by reducing their apoptosis, thus promoting tumor progression.

The MAPK/Erk signaling pathway is associated with cell proliferation, differentiation, and apoptosis. It has also been identified as a classical antiapoptotic pathway in endothelial cells [57]. Additionally, hypoxia-related angiogenesis is reportedly regulated by the Erk pathway [58]. Our previous work showed that Erk is involved in the generation of M-MDSCs in tumor-bearing mice [50]. In the present study, it was revealed that with tumor progression, the upregulation of G-CSF can activate Erk signaling in G-MDSCs to resist apoptosis and promote their survival. Consequently, these results contribute to furthering our understanding of the persistence of G-MDSCs under tumor conditions.

CREB is a nuclear transcription factor that regulates the transcription of several genes that contain CRE sites in their promoters. Transcription begins once CREB is activated and the CREB-binding protein has been recruited [59,60]. Several reports indicate that the activity of CREB is correlated with various intracellular processes. In neurons, CREB is involved in the regulation of neurogenesis and neuronal plasticity [61,62]. Recent data have also suggested that the aberrant expression of CREB is correlated with tumor grade and stage [63]. However, the relationship between CREB and the survival of immunosuppressive cells under tumor conditions remains unclear. In this study, CREB was associated with the survival of G-MDSCs under tumor conditions. Moreover, the activation of CREB reduced the apoptosis of G-MDSCs, thus revealing the role of CREB in the tumor immunosuppressive microenvironment.

In summary, our study demonstrates that G-CSF activates a pro-survival transcriptional program through the Erk/CREB signaling axis, thereby sustaining the survival of G-MDSCs. In a G-CSF-enriched tumor microenvironment, G-MDSCs driven by this mechanism form a functionally distinct subset characterized by a unique dependency on this pathway, which sets them apart from classical tumor-derived MDSCs that exhibit greater heterogeneity and are co-regulated by multiple signaling pathways. Furthermore, experimental validation confirms that inhibiting this axis effectively promotes G-MDSC apoptosis and suppresses tumor progression. However, it should be kept in mind that the proposed G-CSF-G-MDSC axis was investigated primarily in the B16F10 melanoma model. Given the substantial heterogeneity in G-CSF production and myeloid responses across tumor types, whether this mechanism is broadly applicable to other malignancies remains to be determined, and validation in additional tumor models will be important in future studies. Moreover, we cannot rule out the direct effects of these inhibitors on tumor cells or other immune populations, and further studies are warranted to dissect their cell-type-specific contributions. In conclusion, these findings reveal a context-dependent mechanism of MDSC maintenance and establish a theoretical foundation for precision therapies targeting tumors with elevated G-CSF expression. Consequently, developing targeted strategies based on this pathway, such as the delivery of CREB inhibitors using advanced liposomes or nanoemulsion systems [64,65], holds significant translational potential in the field of cancer therapeutics.

## 5. Conclusions

In conclusion, we demonstrated that tumor-derived G-CSF sustains G-MDSC survival by activating the ERK/CREB signaling axis, thereby promoting tumor progression. Disruption of this pathway enhances G-MDSC apoptosis and restricts tumor growth, highlighting the G-CSF/ERK/CREB axis as a potential therapeutic target. Future studies in additional tumor models and clinical settings are needed to validate its broader applicability.

## Data Availability

The original contributions presented in this study are included in the article/Supplementary Materials. Further inquiries can be directed to the corresponding authors.

## Supplementary Materials

The following supporting information can be downloaded at: https://www.mdpi.com/article/10.3390/biology15171511/s1, Figure S1: The original Western blotting images; Figure S2: The proliferation of G-MDSCs in the peripheral blood of mice bearing tumor at different stages did not change; Figure S3: CXCL10 and CXCL13 did not affect the apoptosis of G-MDSCs. Table S1: Primer sequences of qPCR; Table S2: Primer sequences of shRNAs.

## Abbreviations

The following abbreviations are used in this manuscript:

MDSCs	Myeloid-derived suppressor cells
G-CSF	Granulocyte colony-stimulating factor
Arg1	Arginase-1
iNOS	Inducible nitric oxide synthase
G-MDSCs	Granulocytic MDSCs
M-MDSCs	Mononuclear MDSCs
Erk1/2	Extracellular-signal-regulated kinases 1 and 2
MAPK	Mitogen-activated protein kinase
CREB	cAMP response element-binding protein
