# Supplementary material for: The Erk/CREB Signaling Axis Mediates G-CSF-Promoted G-MDSC Survival in the Peripheral Blood of Melanoma-Bearing Mice
Source: Biology (Basel). 2026 Sep 3;15(17):1511. doi: 10.3390/biology15171511 (PMC13564542; doi:10.3390/biology15171511)
Supplement: Supplementary file 1 [file biology-15-01511-s001.zip › biology-4471877-supplementary.pdf]

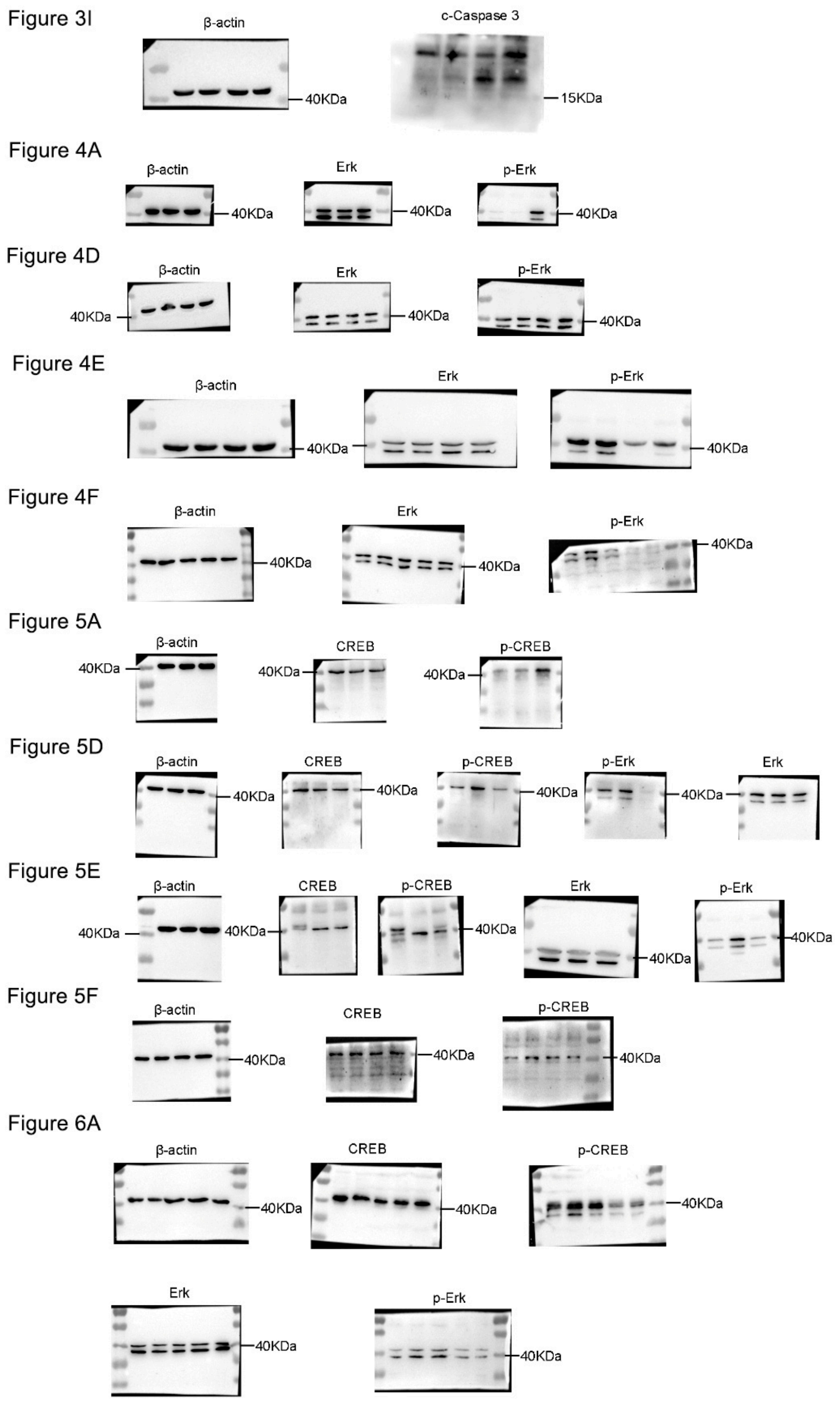

Figure S1. The original Western blotting images.

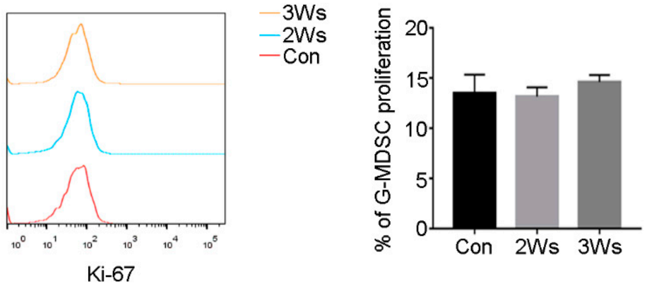

Figure S2. The proliferation of G-MDSCs in the peripheral blood of mice bearing tumor at different stages did not change. The proliferation of G-MDSCs in peripheral blood of mice bearing tumor at different stages was analysed by flow cytometry. Results are mean ± SEM. Unpaired t-test was used to determine significance.

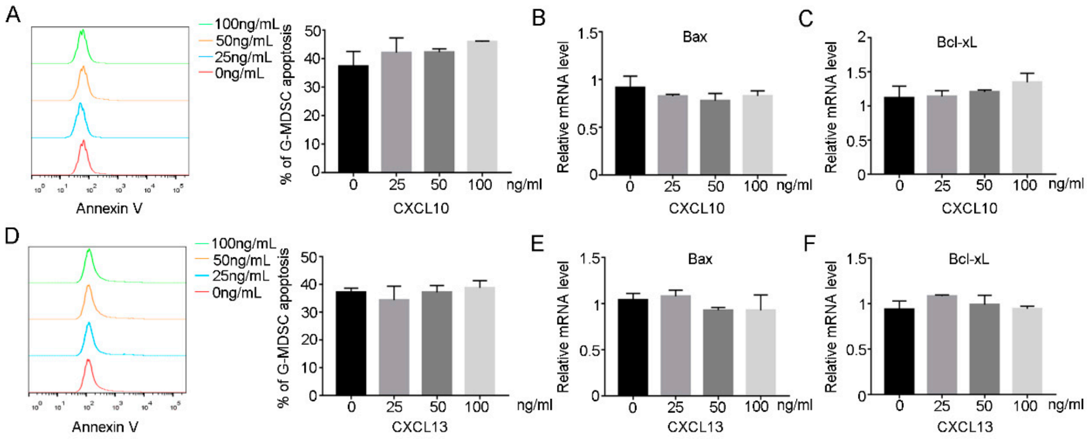

Figure S3. CXCL10 and CXCL13 did not affect the apoptosis of G-MDSCs

(A) The apoptosis of G-MDSCs in the bone marrow of normal mice was analysed by flow cytometry after cultured in vitro with different concentrations of CXCL10. (B-C) The expression levels of Bax and Bcl-xL in normal mouse bone marrow G-MDSCs were analysed by real-time PCR after incubation with different concentrations of CXCL10. (D) The apoptosis of G-MDSCs in the bone marrow of normal mice was analysed by flow cytometry after cultured in vitro with different concentrations of CXCL13. (E-F) The expression levels of Bax and Bcl-xL in normal mouse bone marrow G-MDSCs were analysed by real-time PCR after incubation with different concentrations of CXCL13. Results are mean ± SEM. Unpaired t-test was used to determine significance.

Table S1

Primer sequences of qPCR

Gene	Forward Primer(5'-3')	Reverse Primer(5'-3')
β-actin	AACAGTCCGCCTAGAAGCAC	CGTTGACATCCGTAAAGACC
Bax	CTGATGGCAACTTCAACTG	ATCTTCTTCCAGATGGTGAG
Bcl-xL	GGAGAGCGTTCAGTGATC	AGGTGGTCATTCAGATAGGT
G-CSF	CTGGAGCAAGTGAGGAAGATC	TGGAAGGCAGAAGTGAAGG
G-CSFR	GATAGAACCTAACGGGAACA	GTGACGGAGAAGGAGTGG
CXCL10	GCCTCATCCTGCTGGGTCTG	TTTGGCTAAACGCTTTCATT
CXCL13	GCAACGCTGCTTCTCCTCCT	GTAACTTGAATCCGATCTATG

Table S2

Primer sequences of shRNAs

Primer name	Forward Primer(5'-3')	Reverse Primer(5'-3')
shControl	GATCCCCTTCTCCGAA<br>CGTGTCACGTTTCAAGAG<br>AACGTGACACGTTCGGAG<br>AATTTTTC	TCGAGAAAAATTCT<br>CCGAACGTGTCACGTTCT<br>CTTGAAACGTGACACGTTC<br>GGAGAAGGG
shG-CSF#1	CGCGTCCCCGGTAGG<br>AGAGGACAGATAATTCAA<br>GAGATTATCTGTCCTCTCCTA<br>CCTTTTTGGAAAT	CGATTTCCAAAAAGG<br>TAGGAGAGGACAGATAA<br>TCTCTTGAATTATCTGTCCT<br>CTCCTACCGGGGA
shG-CSF#2	CGCGTCCCCGGTGCC<br>ATTCTCTACTAATTTCAA<br>GAGAATTAGTAGAGAATGGC<br>ACCTTTTTGGAAAT	CGATTTCCAAAAAGG<br>TGCCATTCTCTACTAATT<br>CTCTTGAAATTAGTAGAGAA<br>TGGCACCGGGGA
